# Improving lupeol production in yeast by recruiting pathway genes from different organisms

**DOI:** 10.1038/s41598-019-39497-4

**Published:** 2019-02-28

**Authors:** Weibo Qiao, Zilin Zhou, Qin Liang, Isidore Mosongo, Changfu Li, Yansheng Zhang

**Affiliations:** 1 CAS Key Laboratory of Plant Germplasm Enhancement and Specialty Agriculture, Wuhan Botanical Garden, Chinese Academy of Sciences, Wuhan, 430074 China; 2 University of Chinese Academy of Sciences, Beijing, 100049 China; 3 Shanghai Key Laboratory of Bio-Energy Crops, Research Center for Natural Products, School of Life Sciences, Shanghai University, Shanghai, 200444 China

## Abstract

Lupeol is a pentacyclic triterpene that shows a variety of pharmacological properties. Compared to engineering the production of sesquiterpenes and diterpenes, it is much more challenging to engineer the biosynthesis of triterpenes in microbial platforms. This study showed our efforts on engineering the triterpene pathway in *Escherichia coli* and *Saccharomyces cerevisiae* cells by recruiting the codon-optimized three lupeol pathway genes from different organisms. By comparing their activities with their respective counterparts, the squalene synthase from *Thermosynechococcus elongates* (tSQS), the squalene epoxidase from *Rattus norvegicus* (rSE) and the lupeol synthase from *Olea europaea* (OeLUP) were introduced into *E*. *coli* BL21(DE3), a break-through from zero was observed for lupeol biosynthesis in a prokaryotic host. We also assessed the lupeol pathway under two different yeast backgrounds-WAT11 and EPY300, and have found that the engineered strains based on EPY300, named E*CHHOe*, processed the best lupeol-producing ability with the maximum lupeol titer being 200.1 mg l^−1^ at 30 °C in a 72 h-flask culture, which so far was the highest amount of lupeol obtained by a microbial system and provides a basis for further industrial application of lupeol in the future.

## Introduction

Lupeol is a commercially important pentacyclic triterpene, which exists in a variety of vegetables, fruits and medicinal plants^1–3^. This compound has attracted increasing attention, due to its various beneficial effects on human health (such as anti-tumor, anti-diabetes, and anti-inflammation, and medical treatment of arthritis, hepatic and renal toxicity)^4–8^. Its precursor squalene has also been used as an antioxidant agent and a potential biofuel^9,10^.

**Figure 1:**
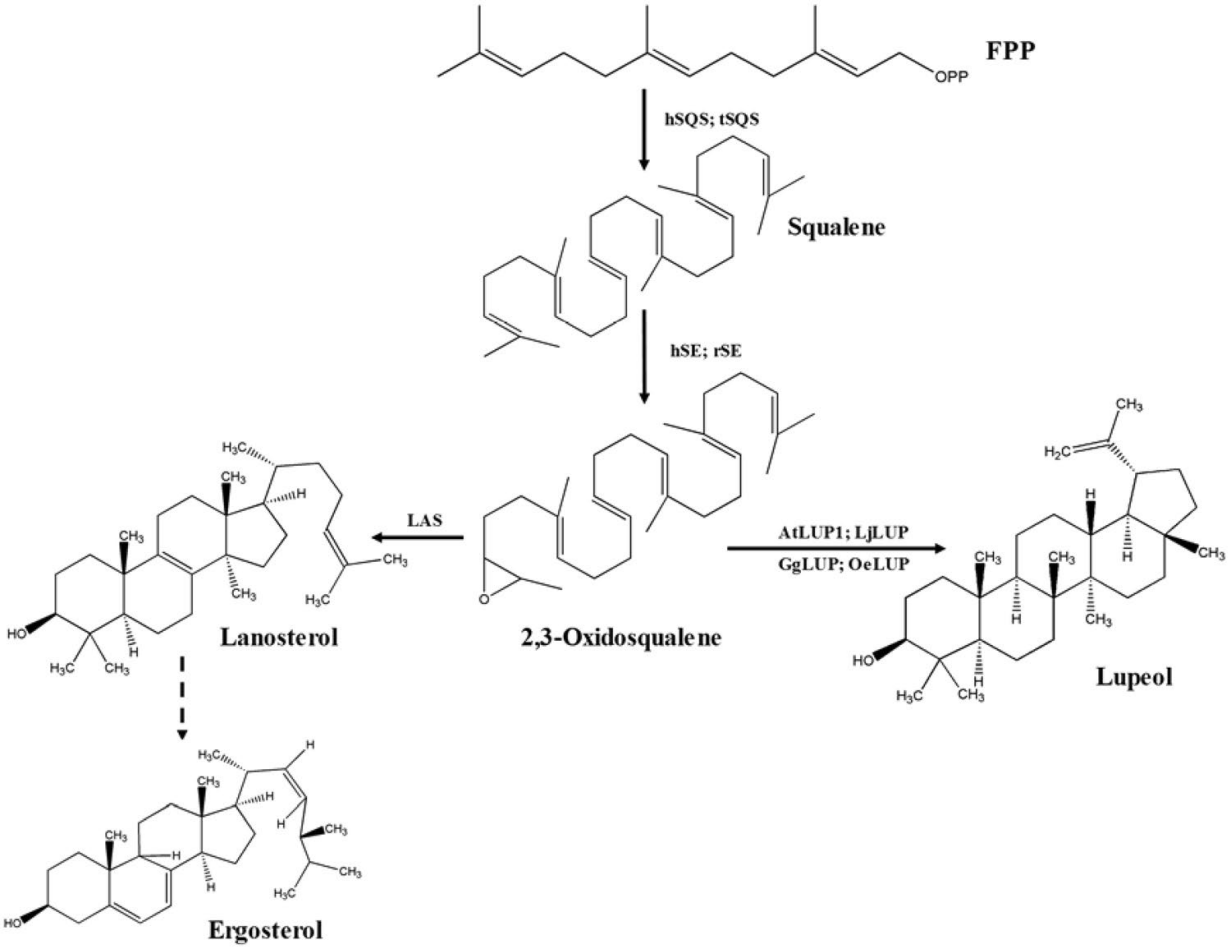
The lupeol pathway starting from FPP. The pathway enzyme variants derived from different organisms were as follows: tSQS (GenBank accession no. NP_681887), SQS from *T*. *elongatus*; hSQS (GenBank accession no. NP_004453), SQS from *H*. *sapiens*; rSE (GenBank accession no. NP_058832), SE from *R*. *norvegicus*; hSE (GenBank accession no. NP_003120), SE from *H*. *sapiens*; AtLUP1 (GenBank accession no. AEE36187), LUP from *A*. *thaliana*; LjLUP (GenBank accession no. AB181245), LUP from *L*. *japonicas*; GgLUP (GenBank accession no. AB116228), LUP from *G*. *glabra*; and OeLUP (GenBank accession no. AB025343), LUP from *O*. *europaea*; LAS, lanosterol synthase.

Starting from farnesyl diphosphate (FPP), the biosynthesis of lupeol begins with the condensation of two molecules of FPP to squalene by squalene synthase (EC 2.5.1.21; SQS) (Fig. 1). Squalene is then oxidized to 2,3-oxidosqualene by a membrane-bound monooxygenase, squalene epoxidase (EC 1.14.14.17; SE). After the oxidosqualene formation, there is a branch point for either sterol (e.g. ergosterol) or lupeol biosynthesis. By the action of lupeol synthase (EC 5.4.99.41; LUP), oxidosqualene is cyclized to form lupeol (Fig. 1). Genes encoding the aforementioned lupeol pathway enzymes (SQS, SE, and LUP) have been identified from various organisms. For example, the LUP-encoding genes have been cloned, and biochemically characterized from *Arabidopsis thaliana*^11^, *Lotus japonicas*^12^, *Glycyrrhiza glabra*^13^ and *Olea europaea*^13^. In many cases, lupeol naturally occurs at very low levels in plant tissues^14^, which has seriously limited its industrial application. For these reasons, engineering lupeol production in microbes is an attractive alternative to extraction from plant sources. The *Escherichia coli* and *Saccharomyces cerevisiae* are two well-established ‘microbial factories’, which have been widely used for generation of many types of compounds, including triterpenes^15,16^. Due to the lack of the SE enzyme (Fig. 1), *E*. *coli* host does not possess any sterol pathway, and the SE expression is definitely needed to engineer triterpene production in this organism. To the best of our knowledge, there are no any reports regarding the production of lupeol in *E*. *coli*. In contrast, the metabolic flux up to oxidosqualene naturally exists in *S*. *cerevisiae*, and in fact lupeol biosynthesis was ever successfully engineered in a yeast strain called NK2-LUP, however, the lupeol amount (8.2 mg l^−1^) yielded from that yeast strain remains pretty low^17^.

With an aim to improve lupeol biosynthesis in microorganisms, this study presented the following efforts: Firstly, the three lupeol pathway genes (*SQS*, *SE* and *LUP*) derived from different organisms were codon-optimized based on *E*. *coli* or *S*. *cerevisiae* preference and their *in vivo* performance with regard to their enzymatic products was evaluated; Secondly, by recruiting the better pathway candidate genes, the lupeol pathway was reconstituted, and the utility of this recruited pathway was evaluated under two different yeast strains-WAT11^18^ and EPY300^19^. For the EPY300 strain, the carbon flux up to FPP, which is the precursor for lupeol biosynthesis (Fig. 1), was genetically enhanced by overexpressing precursor genes^19^. Finally, a highly lupeol-producing yeast strain E*CHHOe* was established by this study with the lupeol titer reaching 200.1 mg l^−1^, which was 24.4 folds higher than that produced by the previously engineered yeast strain NK2-LUP^17^. Moreover, a serious bottleneck of engineering lupeol biosynthesis in *E*. *coli* was firstly discovered in this study, which would provide a valuable guidance for a prokaryotic production of lupeol in the future work.

## Results

### Engineering the lupeol pathway in *E*. *coli*

**Figure 2:**
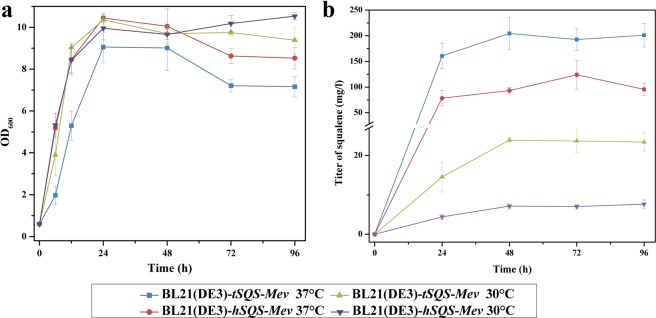
The tSQS displayed a higher activity than the hSQS in *E*. *coli*. (**a**) Growth properties of the *SQS*-transformed strains; (**b**) Squalene yields produced by the *SQS*-transformed strains. The engineered *E*. *coli* BL21(DE3)-*SQS*-*Mev* strains were cultivated at either 30 °C or 37 °C, and cells were respectively harvested at different time intervals for the product analysis.

In the lupeol pathway starting from FPP, the SQS is the first enzyme (Fig. 1). The SQSs from *Thermosynechococcus elongatus* (tSQS) and *Homo sapiens* (hSQS) were chosen in this study, as they showed a relatively higher squalene-producing activity compared to the other SQSs reported so far^20^. The gene sequences of *tSQS* and *hSQS* were manually synthesized based on *E*. *coli* codon usage preference and individually expressed in an *E*. *coli* strain, BL21(DE3). However, both tSQS and hSQS initially led to very low levels of squalene (Fig. S1). We suspected that this low production of squalene was probably due to the limited FPP precursor supply in the transgenic *E*. *coli* cells. To increase the FPP supply, the pBbA5c-M-M plasmid, which contains eight mevalonate (MVA) pathway genes from acetyl-coA to FPP^21^ (see the section of Materials and Methods), was co-expressed. Apparently, the simultaneous overexpression of these eight MVA pathway genes resulted in a substantial increase in lupeol production with the tSQS generating more squalene than the hSQS (Fig. S1). The better performance of tSQS relative to hSQS was further confirmed by measuring squalene yields in a time course manner from both 30 °C- and 37 °C-incubated cultures (Fig. 2).

**Figure 3:**
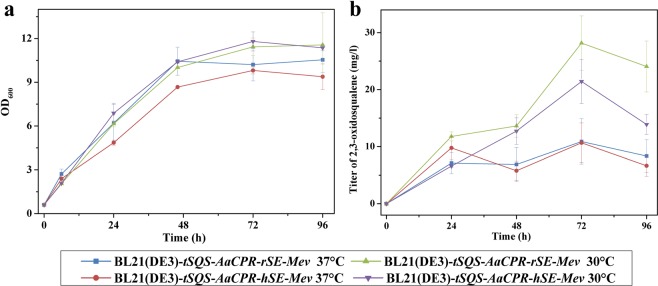
Comparison of the *in vivo* performances between rSE and hSE in *E*. *coli*. (**a**) Growth properties of the *SE*-expressed strains; (**b**) 2,3-Oxidsqualene yields produced by the *SE*-expressed strains. The SEs were expressed in the strain background of BL21(DE3)-*tSQS*-*AaCPR*-*Mev*, and the *SE*-expressed strains were cultured at either 30 °C or 37 °C for four days. During the incubations, the cells were harvested at different time points and used for the analysis.

The next enzyme after squalene synthesis in the lupeol pathway is SE (Fig. 1). SE is a membrane-bound monooxygenase, which makes it difficult to obtain a soluble SE protein in *E*. *coli* cells^22^. Therefore, N-terminal transmembrane domain truncated version of SE was made (see the Materials and Methods section). The *SEs* from *Rattus norvegicus* (*rSE*)^23^ and *H*. *sapiens* (*hSE*)^24^ were individually co-expressed in the presence of the *tSQS* and the eight MVA pathway genes. Because SE is a flavin-dependent monooxygenase, a P450 reductase partner, AaCPR from *Artemisia annua*, was also expressed, which led to the construction of two *E*. *coli* strains of BL21(DE3)-*tSQS*-*AaCPR*-*rSE*-*Mev* and BL21(DE3)-*tSQS*-*AaCPR-hSE*-*Mev*. Both strains were cultured at two temperatures (30 °C and 37 °C), and their 2,3-oxidosqualene yields were compared. Generally, the 30 °C-incubated strains produced more 2,3-oxidosqualene than the 37 °C-cultured ones (Fig. 3). Between the *E*. *coli* strains containing rSE and hSE, there was no significant difference in their 2,3-oxidosqualene yields, indicating that the biochemical efficiencies of rSE and hSE are similar in this *E*. *coli* host.

The last enzyme for lupeol synthesis is LUP (Fig. 1). To screen the LUP variants, the LUPs from *A*. *thaliana* (AtLUP1)^11,25^, *L*. *japonicas* (LjLUP)^12^, *G*. *glabra* (GgLUP)^13^ and *O*. *europaea* (OeLUP)^13^ were investigated. These LUP-encoding genes were initially codon-optimized based on the *E*. *coli* usage preference and individually expressed in the strain of BL21(DE3)-*tSQS*-*AaCPR*-*rSE*-*Mev*. However, only a trace amount of lupeol was observed in the *OeLUP*-expressed strain (Fig. S2), and none was detected by the others, which was probably due to a false folding of these LUPs in *E*. *coli* cells. This hypothesis could be supported by the observation that AtLUP1 and its truncated mutants were exclusively presented as inclusion bodies when they were solely expressed in BL21(DE3) cells (Fig. S3), suggesting that *E*. *coli* might not be a suitable host for functional expression of LUP enzyme.

### Engineering the lupeol pathway in *S*. *cerevisiae*

**Figure 4:**
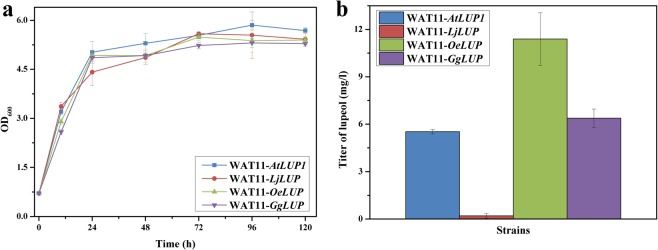
Functional analysis of different organisms-derived LUPs based on the WAT11 yeast strain. (**a**) Growth property; (**b**) Lupeol titer. The *LUP*-expressed strains were cultivated at 30 °C for five days, and both the cells and medium were separately harvested after the 72 h-cultivation for the product analysis.

To examine whether LUP could be functionally expressed in *S*. *cerevisiae*, the aforementioned four LUP genes were re-synthesized following *S*. *cerevisiae* codon usage and initially expressed in a WAT11 yeast strain^18^, which naturally provides the precursor 2,3-oxidosqualene. In comparison with the controls, the expression of each of the four LUPs led to an obviously new peak which displayed the same retention time and the same mass spectrum patterns with those of authentic lupeol standard (Fig. S4), suggesting that LUP could be correctly expressed by *S*. *cerevisiae*. Next, we proceeded to assess the activity of each LUP by measuring lupeol amounts from the *LUP*-transformed yeast cells at different time points. As shown in Fig. 4a, the yeast strains expressing each LUP displayed similar growth rates, while with regard to lupeol yield, the *OeLUP* expression yielded the maximum amount and the expression of *LjLUP* caused the least (Fig. 4b). The functional expression of LUP enzyme in yeast allowed us to proceed to compare the contributions of the *SQS* and *SE* variants to lupeol production in this eukaryotic host.

**Figure 5:**
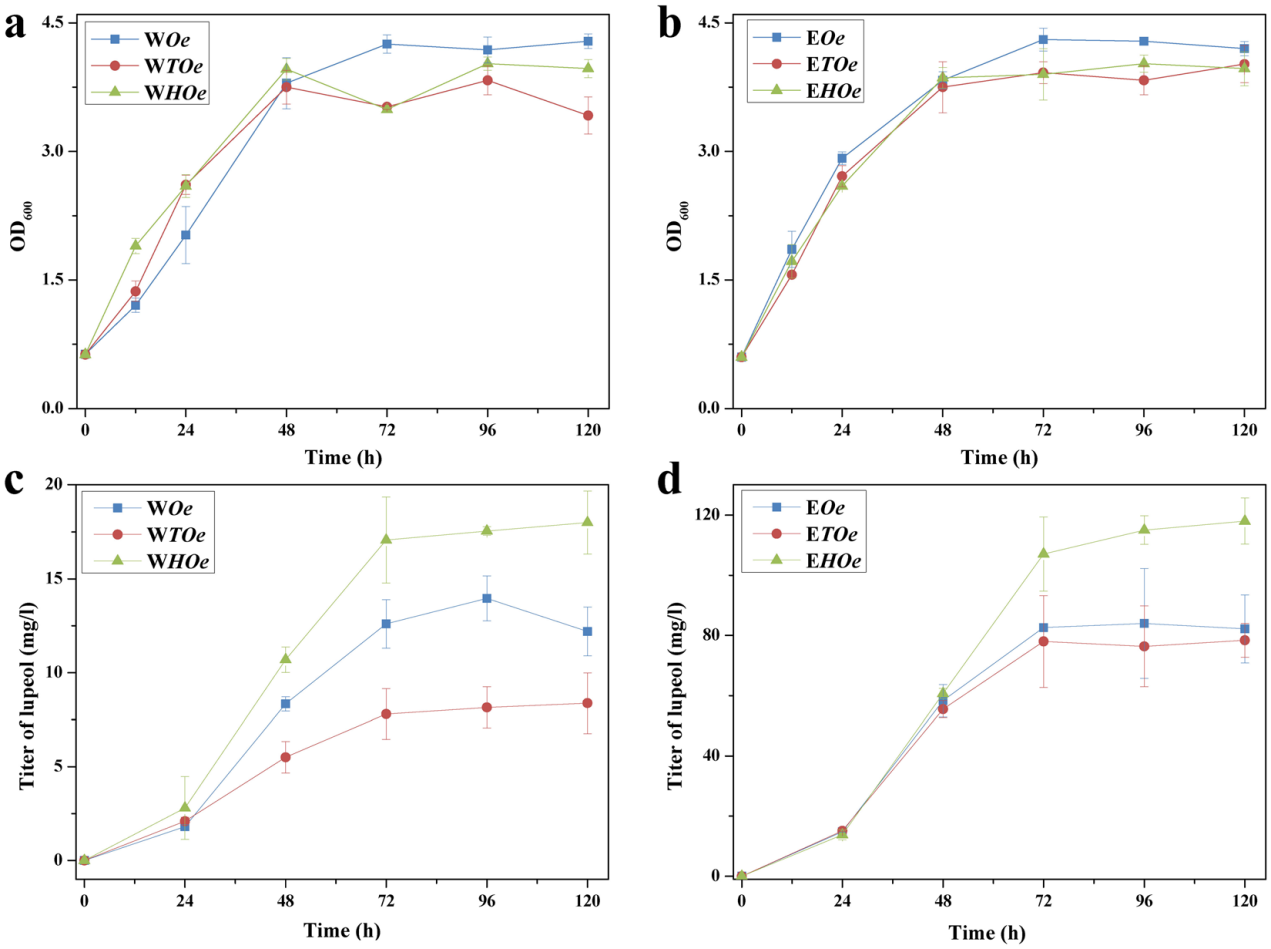
Functional analysis of different organisms-derived SQSs in the presence of the *OeLUP* under two different yeast strains (WAT11 and EPY300). The growth properties were shown for the WAT11-(**a**) and EPY300-(**b**) based engineered yeast strains. The lupeol yields were shown for the WAT11-(**c**) and EPY300-(**d**) based engineered yeast strains. The engineered strains were cultivated at 30 °C for five days, and both the cells and medium were separately harvested at different time intervals for the product analysis.

To compare the *in vivo* performances between tSQS and hSQS, they were individually co-expressed with OeLUP under two different yeast backgrounds (WAT11 and EPY300). As shown in Fig. S5, in either yeast background, tSQS generated a little bit higher squalene than hSQS, which trend was consistent with that obtained from the *E*. *coli* expression system (Fig. 2). Interestingly, although hSQS showed a relatively lower squalene-producing activity, it conversely generated significantly higher levels of lupeol than tSQS in both yeast backgrounds at all the time points (Fig. 5), suggesting that hSQS was more active in the flux toward lupeol production. The effects of the *SQS* expressions on yeast endogenous ergosterol yields were also examined using a 72 h-cultivated yeast cells. In both WAT11 and EPY300 backgrounds, the expression of either *tSQS* or *hSQS* increased ergosterol yields, this improvement was more obvious under the EPY300 background (Fig. S5).

**Figure 6:**
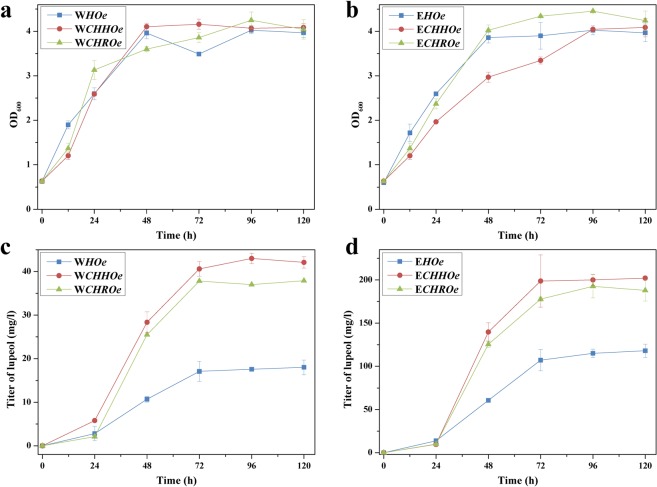
Functional analysis of different organisms-derived SEs in the presence of both OeLUP and hSQS under the two yeast backgrounds. The growth properties were shown for the WAT11-(**a**) and EPY300-(**b**) based engineered yeast strains. The lupeol yields were shown for the WAT11-(**c**) and EPY300-(**d**) based engineered yeast strains. The engineered strains were cultivated at 30 °C for 120 h, and both the cells and medium were separately harvested at different time intervals for the product analysis.

Based on the above experiments that identified hSQS and OeLUP as the better components in the lupeol pathway, they were co-expressed in both WAT11 and EPY300 backgrounds, resulting in two basis strains, named W*HOe* and E*HOe*, respectively. To evaluate the *SE* variants (*rSE* and *hSE*), they were individually expressed in these two basis strains. As an expression partner of SE, the reductase AaCPR was also co-expressed. It should be noted that 2,3-oxidosqualene product was not detectable in all the engineered yeasts, suggesting that it was efficiently converted to downstream end products like lupeol. Compared to the basis strains (W*HOe* and E*HOe*), the strains expressing either rSE (W*CHROe* and E*CHROe*) or hSE (W*CHHOe* and E*CHHOe*) produced 1.6–2.6 fold higher lupeol levels at all the time points after 48 h of cultivation (Fig. 6), suggesting that the transformed SEs were functional in yeast cells. The increase in lupeol production was accompanied with an obvious declining in endogenous ergosterol accumulation (Fig. S5), which indicated that the flux through the ergosterol pathway was deviated to lupeol production by the SE expression. There was no dramatic difference in lupeol yields between the *rSE*- and *hSE*-expressed yeast strains (Fig. 6), indicating of their similar activities. This data was also consistent with that generated from our *E*. *coli* experiments (Fig. 3). Overall, the lupeol amount produced by the EPY300-based yeast strains was 4.6–9.4 fold higher than that produced by the WAT11-based chassis. This data was reasonable as in the EPY300 strain the precursor flux toward FPP was strengthened^19^.

## Discussion

In general, the biosyntheses of triterpenoids and sterols compete for the same precursor 2,3-oxidosqualene, after which they are branched through different cyclization reactions by unique oxidosqualene cyclases (OSCs). Due to the importance of sterols for cell growth^26^, it is challenging to engineer the carbon flux predominantly transferred to triterpenoids biosynthesis in eukaryotes. On the other hand, the prokaryote *E*. *coli* does not possess any 2,3-oxidosqualene synthase and accordingly no sterols biosynthesis has been reported by this organism. The absence of sterol-producing ability of *E*. *coli* might provide a potential for high-titer triterpenoids production, hence in this study, firstly, we made efforts to engineer lupeol biosynthesis in *E*. *coli*.

Three lupeol pathway genes (*SQS*, *SE* and *LUP*) originally identified from different organisms were manually synthesized and their *in vivo* performances were compared. Consistent with their *in vitro* biochemical characterizations previously reported^23,24^, with regard to squalene production, tSQS performed better than hSQS *in vivo* in both *E*. *coli* and yeast systems of this study (Figs 2 and S5). SE is a membrane-localized monooxygenase, which makes it difficult to obtain a soluble SE protein in *E*. *coli* cells. Fortunately, a successful expression of SE in *E*. *coli* has previously been realized by deleting or swapping its N-terminal transmembrane domains^23,24^. Using the same strategy in this study, active rSE and hSE were also successfully expressed in *E*. *coli*, and a slightly higher amount of 2,3-oxidosqualene was produced by rSE compared to hSE. Further attempts to express the *LUP* genes in *E*. *coli* failed to produce lupeol, except that only a trace amount of lupeol was observed in the *Oelup-*expressed *E*. *coli* cells. We presumed that the deficiency of lupeol synthesis in *E*. *coli* could be due to the incorrect folding of LUP proteins. This hypothesis was supported by the fact that the sole expression of *AtLUP1* in *E*. *coli* cells formed the LUP protein almost completely as inclusion bodies (Fig. S3). This data might explain why a successful production of lupeol in *E*. *coli* has never been reported before this study. Using an online software (http://harrier.nagahama-i-bio.ac.jp/sosui/sosui_submit.html), bioinformatics analysis predicted that several transmembrane domains exist in the N-terminus of the LUPs (Fig. S3), and the presence of membrane spanning segments probably decrease water-solubility of protein itself ^27^. We made efforts to get soluble LUP proteins by deleting their N-terminal transmembrane domains. Unfortunately, these deletions did not rescue the LUP expression problems in *E*. *coli* host. Further protein engineering of these LUPs is required to facilitate the functional production of lupeol in *E*. *coli*.

Because of no much success of producing lupeol in *E*. *coli* host, we switched to engineer the lupeol pathway in *S*. *cerevisiae*. Lupeol production was apparently observed by expressing each LUP enzyme in WAT11 strain, suggesting that LUP was functionally expressed. Among the LUPs tested here, OeLUP was discovered to produce the highest amount of lupeol and therefore it was selected as the LUP enzyme in the recruited pathway. The *OeLUP*-expressed yeast strains were then subjected to screen the *SQS* and *SE* candidates. Two different yeast strains with different backgrounds were used as the chassis for this experiment. Interestingly, relative to tSQS, hSQS, which produced the less squalene, generated more lupeol in both yeast backgrounds. A similar case was also reported for the co-expression of different SQSs with a β-amyrin synthase in yeast^28^. We could not explain this inconsistency, one possibility that could attribute to this disparity is that the hSQS associated with its downstream enzymes in a more tuneful context to direct the carbon flux. Compared to the *SQS* expression, additional expression of the SE, especially in the WAT11 background, caused a significantly larger increase in lupeol production (Fig. S5), which indicated that SE might be a rate-limiting enzyme in the lupeol pathway. With respect to yeast strain selection, EPY300 generally produced more than 4-fold higher level of squalene and 5-fold of lupeol than WAT11, which is reasonable because the carbon flux up to FPP was enhanced in EPY300^19^. The maximum amount (200.1 mg l^−1^) of lupeol obtained by the EPY300-based engineered strains (named E*CHHOe*) was 24.4 fold higher than that (8.2 mg l^−1^) produced by the previously engineered yeast strain NK2-LUP^17^. The difference in lupeol yields between this work and that study was most likely due to the different yeast strains used. Moreover, the rate-limiting enzyme SE was not overexpressed in the yeast strain NK2-LUP, which may partially account for the relatively lower production of lupeol as well. It was noteworthy that the ergosterol was kept at relative low levels in both engineered WAT11 and EPY300 strains. The generation of ergosterol seemed not to be significantly affected by the exogenously invaded pathway, confirming that ergosterol was essential for yeast growth and thus its metabolic flux was tightly regulated.

## Conclusions

Based on *E*. *coli* and *S*. *cerevisiae* hosts, the three lupeol pathway genes (*SQS*, *SE* and *LUP*) derived from different organisms were systematically evaluated for their *in vivo* performances. The *tSQS* from *T*. *elongatus*, the *rSE* from *R*. *norvegicus* and the *OeLUP* from *O*. *europaea* all showed relatively higher activities than their respective counterparts, and thus were finally selected to reconstitute the lupeol pathway in this study. The reconstituted lupeol pathway was then transferred into two different yeast strains of WAT11 and EPY300 to compare their lupeol-producing abilities. EPY300 was shown to be a potentially idea strain for lupeol production, and it produced 4.6–9.4 fold higher lupeol than WAT11. Taken together, by screening the different lupeol pathway enzyme variants and assessing two different yeast hosts, a highly lupeol-producing yeast strain, named E*CHHOe* here, was obtained with the maximum lupeol titer after a 72 h-flask cultivation reaching 200.1 mg l^−1^, which was 24.4 folds higher than that produced by a previously reported strain that was the only engineered lupeol-producing yeast before this study.

## Materials and Methods

### Plasmids construction

All the plasmids constructed from this study were shown in Table S1. For *SQS* gene cloning, the *tSQS* from *T*. *elongatus* and *hSQS* from *H*. *sapiens* were chosen. For *SE* gene cloning, the *rSE* from *R*. *norvegicus* and the *hSE* from *H*. *sapiens* were selected and their transmembrane domains were deleted when they were synthesized by Genewiz Inc. (Suzhou, Jiangsu, China) (For the rSE, the amino acid residues from 1 to 98 were deleted with Phe223 being mutated to Ala223; for the hSE, the amino acid residues from 1 to 53 and 517 to 574 were truncated). For *LUP* gene cloning, the *AtLUP1* from *A*. *thaliana*, the *LjLUP* from *L*. *japonicas*, the *GgLUP* from *G*. *glabra* and the *OeLUP* from *O*. *europaea* were selected.

For optimal expressions in *E*. *coli* host, all of the selected genes were respectively codon-optimized based on *E*. *coli* codon usage preference. The plasmid pCW-CT, which contains a cytochrome P450 reductase (CPR) encoding gene *AaCPR* and the costunolide pathway genes (*GAS*, *GAO* and *COS*), was provided by Liu’s group^29^ and used for *SQS* and *SE* gene cloning and expression in *E*. *coli*. Each synthesized *SQS* gene was cloned into pCW-CT by *Nde* I/*Sac* II digestion to replace the costunolide pathway genes which were previously engineered in it, yielding the constructs pCW-*tSQS* and pCW-*hSQS*. To construct a *tSQS*-*SE* co-expression system in *E*. *coli*, the *tSQS* and *SE* were cloned into pCW-CT through successive enzyme digestions and ligations under *Xba* I/*Sac* II and *Nde* I/*Xba* I sites with the costunolide pathway genes being replaced, resulting in the constructs of pCW-*tSQS*-*AaCPR*-*rSE* and pCW-*tSQS*-*AaCPR*-*hSE*. The plasmid pBbA5c-M-M, which contains eight MVA pathway genes in converting acetyl-CoA to FPP, was originally made by Jay Keasling’s group^21^. The plasmid pET-30a (Stratagene, La Jolla, CA, USA) was used for *LUP* gene cloning and expression in *E*. *coli*.

Similarly, for optimal expression in yeast hosts, all of the selected sequences were also individually codon-optimized according to *S*. *cerevisiae* codon preference. The plasmid pESC-Leu2d-*AaCPR* was obtained from Ro’s group^30^ and used for expressing the *SQS* or *SE* gene in yeast. The *SQS* was cloned into pESC-Leu2d-*AaCPR* under *Not* I/*Pac* I sites, yielding the constructs pESC-Leu2d-*AaCPR*-*tSQS* and pESC-Leu2d-*AaCPR*-*hSQS*. The *SE* was inserted into a yeast expression vector pESC-Ura (Stratagene) with *Bam*H I and *Nhe* I digests to get the constructs pESC-*Ura*-*rSE* and *pESC-Ura-hSE*. To construct the *hSQS*-*SE* co-expression system in yeast, both pESC-Leu2d-*AaCPR*-*hSQS* and pESC-Ura-*SE* were digested with *Pac* I/*Sma* I, yielding the construct pESC-Leu2d-*AaCPR*-*hSQS*-*rSE* and pESC-Leu2d-*AaCPR*-*hSQS*-*hSE*. The *LUP* genes were individually cloned into the yeast expression vectors pESC-His (Stratagene) or pESC-Ura, leading to the constructs of pESC-His-*LjLUP*, pESC-His-*GgLUP*, pESC-His-*AtLUP1* and pESC-Ura-*OeLUP*.

### Expression in *E*. *coli* and metabolite extraction

An *E*. *coli* strain BL21(DE3) (Novagen, USA) was used as the host in this study. The constructed expression vectors were transformed into BL21(DE3) cells and the descriptions regarding to the transgenic *E*. *coli* strains are shown in Table S2. For protein expression, cells were firstly cultivated at 37 °C in terrific broth (TB) medium with appropriate concentration of antibiotics and 20 mM MgSO_4_ to an optical density (OD) of 0.4–0.6 at 600 nm. Expression was induced by adding a final concentration of 0.4 mM isopropyl thio-β-D-galactoside (IPTG) for 4 days at 30 °C or 37 °C. Thereafter, cells were firstly harvested by centrifugation, washed with 150 mM NaCl solution, and re-suspended in 5 ml of acetone followed by a 5 min-vortex. The crude acetone extracts were centrifuged at 10000 g for 20 min, and clear extract was then evaporated and re-suspended in 1 ml of methyl alcohol for HPLC analysis.

### Expression in *S*. *cerevisiae* and metabolite extraction

Two *S*. *cerevisiae* strains, WAT11^18^ and EPY300^19^, were used as the eukaryotic hosts in this study. The constructed expression plasmids were transformed into the yeast cells and the descriptions regarding to the transgenic yeast strains are shown in Table S3. The yeast cells were respectively cultured at 30 °C for 48 h in appropriate synthetic defined dropout liquid medium containing 2% (w/v) glucose. Then cells were firstly collected by centrifugation, re-suspended using the inducible medium which contained 1.8% (w/v) galactose and 0.2% (w/v) glucose, and diluted to the OD_600_ reaching 0.4. At the same time, a final concentration of 100 mM 4-(2-Hydroxyethyl)-1-piperazineethanesulfonic acid (HEPES) buffer (pH 7.0–7.5) was added to the inducible medium. For metabolite extraction from the cells, yeast cells were firstly collected by centrifugation, then washed with 5 ml of 150 mM NaCl solution, and finally vacuum-dried. The dried yeast cells were mixed with 0.5 mm glass beads in acetone solution, and subsequently extracted using a SCIENTZ-48 Tissue Lyser (Scientz Biotechnology Co, Ningbo, China) for 15 min. For metabolite extraction from the medium, liquid culture was directly mixed and extracted with ethyl acetate. Finally, both the acetone extracts from the cells and ethyl acetate extracts from the medium were vacuum-evaporated and derivatized with Bis-N,O-(trimethylsilyl) trifluoroacetamide (BSTFA) at 80 °C for 30 min and subjected to Gas Chromatography-Mass Spectrometer (GC-MS) analysis.

### High Performance Liquid Chromatography (HPLC) and GC-MS analyses

HPLC analysis was performed on an LC-20AT instrument (Shimadzu, Kyoto, Japan) using an Inersil ODS-3 C18 column (250 mm × 4.6 mm × 5 μm). The column temperature was maintained at room temperature using methyl alcohol/acetonitrile (50:50, v/v) as the mobile phase at a flow rate of 1.2 ml min^−1^, and the detection wavelength was set at 205 nm.

GC-MS analysis was quantified using an Agilent Technologies 5975 C gas chromatography equipped with a HP-5 MS column (30 m × 0.25 mm × 0.25 μm, Agilent Technologies, Palo Alto, CA, USA). Nitrogen was used as the carrier gas at a flow rate of 1.2 ml min^−1^. The temperatures of the injector and detector were both set at 250 °C. The column temperature was programmed at an initial temperature of 80 °C for 2 min, then increased to 310 °C at a rate of 20 °C min^−1^, and finally held at 300 °C for 15 min. One microliter of sample was injected into the GC column, and analyzed using the SIM-Scan mode within the m/z range of 50–600.

### Ethics Approval and Consent to Participate

This study does not contain any experiment with human participants or animals performed by any of the authors.

## Supplementary information

Additional Files

## Data Availability

All the data analyzed during this study have been included in this article.
