# Supplementary material for: Improving lupeol production in yeast by recruiting pathway genes from different organisms
Source: Sci Rep. 2019 Feb 28;9:2992. doi: 10.1038/s41598-019-39497-4 (PMC6395594; doi:10.1038/s41598-019-39497-4)
Supplement: Supplementary file 1 — Additional Files [file 41598_2019_39497_MOESM1_ESM.pdf]

Additional Files

Improving lupeol production in yeast by recruiting pathway genes from different organisms

Weibo Qiao[1,2], Zilin Zhou[1,2], Qin Liang[1,2], Isidore Mosongo[1,2], Changfu Li[1,3] and Yansheng Zhang[1,3*]

Correspondence: zhangys1@shu.edu.cn; Telephone: 86-21-66130927.

[1] CAS Key Laboratory of Plant Germplasm Enhancement and Specialty Agriculture, Wuhan Botanical Garden, Chinese Academy of Sciences, Wuhan, 430074, China

[2] University of Chinese Academy of Sciences, Beijing, 100049, China

[3] Shanghai Key Laboratory of Bio-Energy Crops, Research Center for Natural Products, School of Life Sciences, Shanghai University, Shanghai 200444, China

Fig. S1 Comparison of the squalene titers by the *SQS*-transformed *E. coli* cells. **a** HPLC profiles showing the squalene peaks (Peak 1) produced by the engineered strains. A, the strain expressing the MVA module alone; B, the strain expressing the *hSQS* alone; C, the strain expressing the *tSQS* alone; D, the strain co-expressing the MVA module and the hSQS; E, the strain co-expressing the MVA module and the tSQS. **b** Quantification of the squalene yield produced by the engineered *E. coli* strains (A-E). The engineered strains were cultivated at 37°C for 48 h, and cells were respectively harvested to measure the produced squalene.

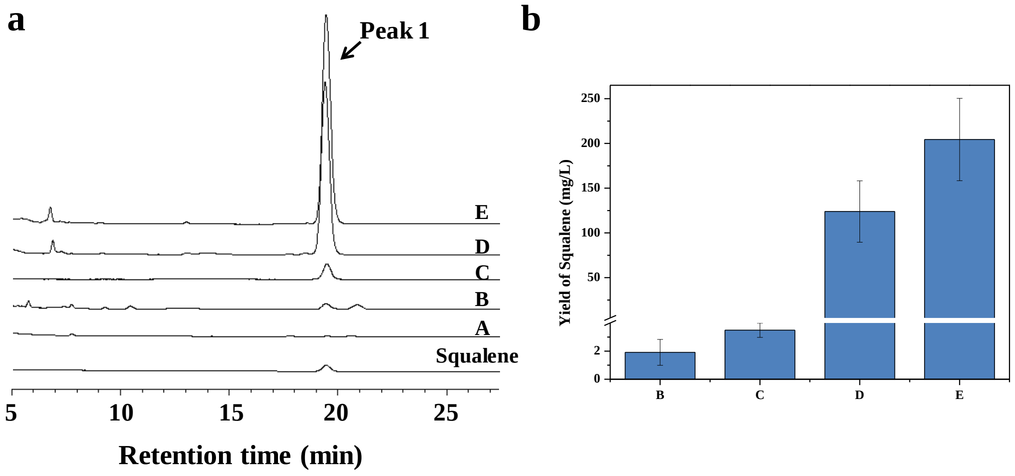

Fig. S2 GC-MS analysis of the lupeol produced by *OeLUP*-expressed *E. coli* cells. **a** GC profiles for lupeol standard, the lupeol product (Peak 3) from the *OeLUP*-expressed *E. coli* strain BL21(DE3)-*tSQS-AaCPR-rSE-Mev-OeLUP*, and the metabolite extracts from the control strain BL21(DE3)-*tSQS-AaCPR-rSE-Mev*. **b** the mass spectrums of lupeol standard and the lupeol product (Peak 3). The transgenic *E. coli* strains were cultivated at 30°C for 48 h, and cells were harvested for the lupeol product analysis.

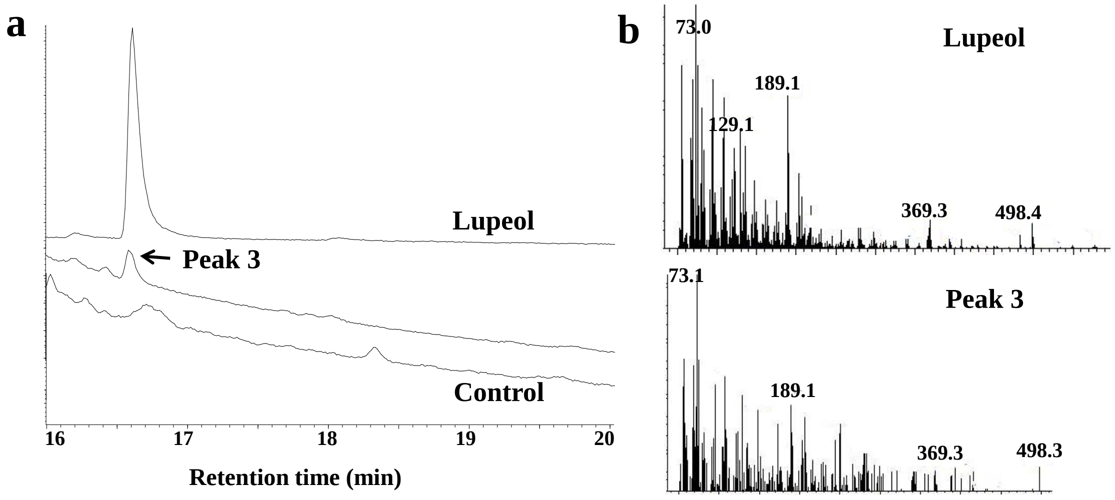

Fig. S3 SDS-PAGE analysis of the recombinant AtLUP1 together with its truncated mutants expressed in *E. coli*. Two truncated forms of AtLUP1 (AtLUP1 (aa 101-757) and AtLUP1 (aa 143-757)) were made by deleting the 1-100 and 1-142 amino acid residues of AtLUP1, respectively. The wild type AtLUP1 as well as its two truncated mutants were cultured at 37°C in lysogeny broth medium with 50 μg ml^{-1} kanamycin to the OD of 0.6 at 600 nm. At this point, a final concentration of 0.4 mM IPTG was then added into the medium to induce the protein expression, and the cells were cultured for 12 hours at 16°C prior to the protein extraction.

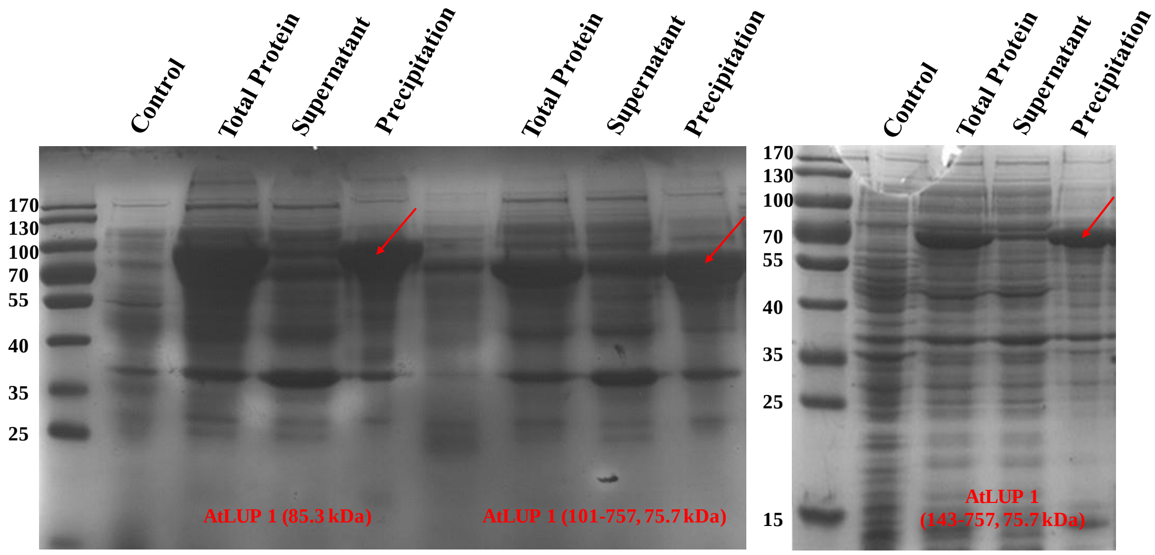

Fig. S4 GC-MS analysis of the lupeol produced by the *LUP*-expressed yeast strain WAT11. **a** GC profiles of the lupeol product (Peak 3); **b** Mass spectrums of the lupeol product (Peak 3) with its chemical standard. The strains of WAT11-*OeLUP*, WAT11-*GgLUP*, WAT11-*AtLUP1*, WAT11-*LjLUP*, and WAT11 were cultivated at 30°C for 72 h, and both the cells and medium were respectively harvested for analyzing the lupeol product.

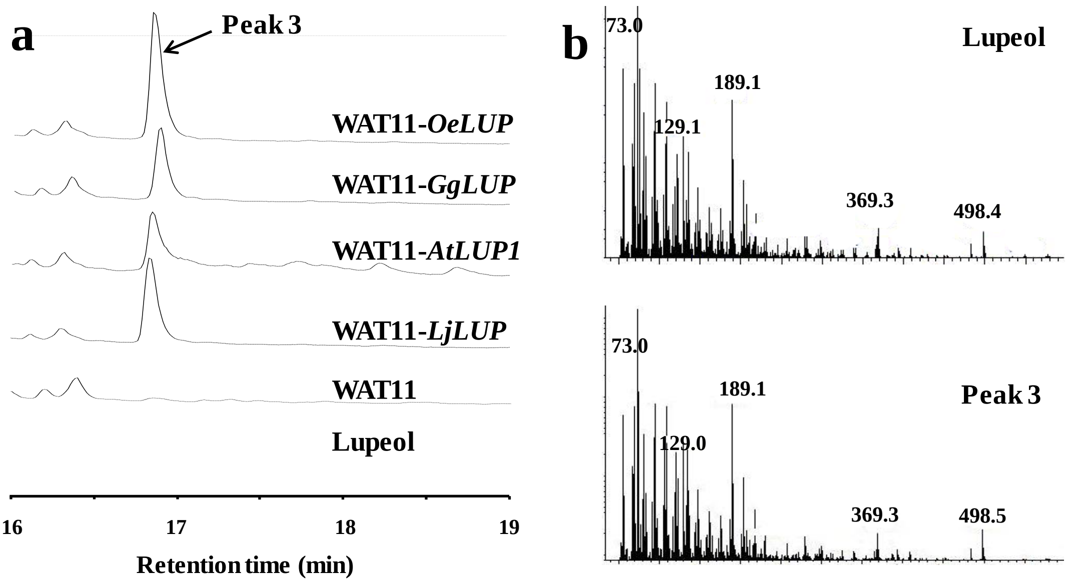

Fig. S5 Comparison of the yields of squalene, ergosterol and lupeol among all the engineered yeast strains. **a** Engineered WAT11 strains; **b** Engineered EPY300 strains. *Oe*, the strain expressing *OeLUP* alone; *Toe*, the strain co-expressing *tSQS* and *OeLUP*; *Hoe*, the strain co-expressing *hSQS* and *OeLUP*; *CHHOe*, the strain co-expressing *hSQS*, *OeLUP* and *hSE*; *CHROe*, the strain co-expressing *hSQS*, *OeLUP* and *rSE*. The engineered strains were cultivated at 30°C, and both the cells and medium were respectively harvested for 72 h to determine the products.

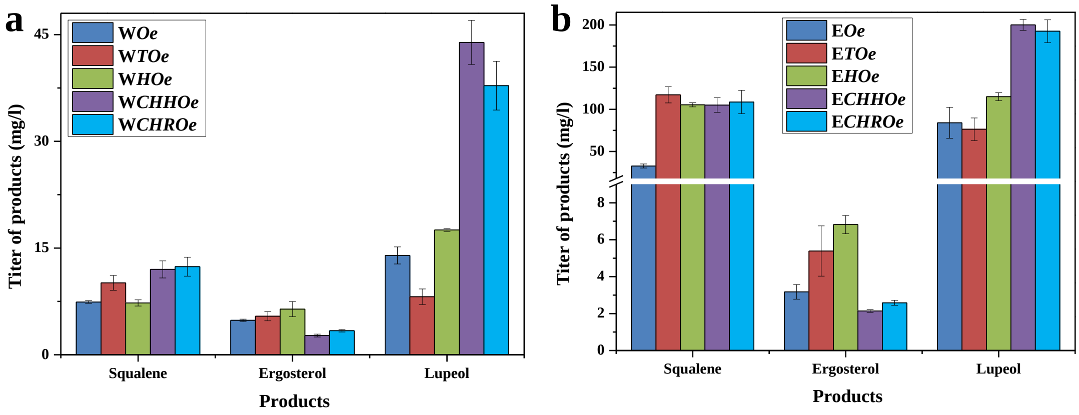

Table S1 The plasmids used in this study

Plasmid name	Plasmid description	Source
**Plasmids used for *E. coli***		
pCW-CT	Expression plasmid pCW-ori harboring *AaCPR*, *GAS*, *GAO* and *COS*; Ap^r	[1]
pCW-*tSQS*	pCW-CT harboring *tSQS*	This study
pCW-*hSQS*	pCW-CT harboring *hSQS*	This study
pCW-*tSQS*-*AaCPR*-*rSE*	pCW-CT harboring *tSQS*, *AaCPR*, and *rSE*	This study
pCW-*tSQS*-*AaCPR*-*hSE*	pCW-CT harboring *tSQS*, *AaCPR*, and *hSE*	This study
pBbA5c-M-M	Expression plasmid pBbA5c harboring *AtoB(o)*-*HMGS(o)*-*HMGR(o)*-*MK(o)*-*PMK (o)*-*PMD*-*Idi*-*IspA*; Cm^r	Addgene [2]
pET-30a	Expression plasmid; Km^r	Stratagene
pET30a-*AtLUP1*	pET-30a harboring *AtLUP1*	This study
pET30a-*LjLUP*	pET-30a harboring *LjLUP*	This study
pET30a-*GgLUP*	pET-30a harboring *GgLUP*	This study
pET30a-*OeLUP*	pET-30a harboring *OeLUP*	This study
**Plasmids used for *S. cerevisiae***		
pESC-Leu2d	Yeast episomal plasmid; Ap^r	[3]
pESC-Leu2d-*AaCPR*	P_{GAL1}*AaCPR*	[3]
pESC-Leu2d-*AaCPR*-*tSQS*	P_{GAL1}*AaCPR,* P_{GAL10}*tSQS*	This study
pESC-Leu2d-*AaCPR*-*hSQS*	P_{GAL1}*AaCPR,* P_{GAL10}*hSQS*	This study
pESC-Leu2d-*AaCPR*-*hSQS*-*rSE*	P_{GAL1}*AaCPR,* P_{GAL10}*hSQS,* P_{GAL1}*rSE*	This study
pESC-Leu2d-*AaCPR*-*hSQS*-*hSE*	P_{GAL1}*AaCPR,* P_{GAL10}*hSQS,* P_{GAL1}*hSE*	This study
pESC-Ura	Yeast episomal plasmid; Ap^r	Stratagene
pESC-Ura-*rSE*	P_{GAL1}*rSE*	This study
pESC-Ura-*hSE*	P_{GAL1}*hSE*	This study
pESC-His	Yeast episomal plasmid; Ap^r	Stratagene
pESC-His-*LjLUP*	P_{GAL1}*LjLUP*	This study
pESC-His-*GgLUP*	P_{GAL1}*GgLUP*	This study
pESC-Ura-*OeLUP*	P_{GAL1}*OeLUP*	This study
pESC-His-*OeLUP*	P_{GAL1}*OeLUP*	This study
pESC-His-*AtLUP1*	P_{GAL1}*AtLUP1*	This study

Table S2 The *E. coli* strains used in this study

**Strain name**	**Plasmids**	**Source**
BL21(DE3)		Novagen
BL21(DE3)-pBbA5c-M-M	pBbA5c-M-M	This study
BL21(DE3)-*tSQS*	pCW-*tSQS*	This study
BL21(DE3)-*hSQS*	pCW-*hSQS*	This study
BL21(DE3)-*tSQS-Mev*	pCW-*tSQS* & pBbA5c-M-M	This study
BL21(DE3)-*hSQS-Mev*	pCW-*hSQS* & pBbA5c-M-M	This study
BL21(DE3)-*tSQS-AaCPR-rSE*	pCW-*tSQS-AaCPR-rSE*	This study
BL21(DE3)-*tSQS-AaCPR-hSE*	pCW-*tSQS-AaCPR-hSE*	This study
BL21(DE3)-*tSQS-AaCPR-rSE-Mev*	pCW-*tSQS-AaCPR-rSE* & pBbA5c-M-M	This study
BL21(DE3)-*tSQS-AaCPR-hSE-Mev*	pCW-*tSQS-AaCPR-hSE* & pBbA5c-M-M	This study
BL21(DE3)-*tSQS-AaCPR-rSE-Mev-AtLUP1*	pCW-*tSQS-AaCPR-rSE* & pBbA5c-M-M & pET30a-*AtLUP1*	This study
BL21(DE3)-*tSQS-AaCPR-rSE-Mev-LjLUP*	pCW-*tSQS-AaCPR-rSE* & pBbA5c-M-M & pET30a-*LjLUP*	This study
BL21(DE3)-*tSQS-AaCPR-rSE-Mev-GgLUP*	pCW-*tSQS-AaCPR-rSE* & pBbA5c-M-M & pET30a-*GgLUP*	This study
BL21(DE3)-*tSQS-AaCPR-rSE-Mev-OeLUP*	pCW-*tSQS-AaCPR-rSE* & pBbA5c-M-M & pET30a-*OeLUP*	This study

Table S3 The yeast strains used in this study

**Strain name**	**Description**	**Source**
WAT11	*MATα*; ade2-1; his3-11,-15; leu2-3,-112; ura3-1; trp1-1	[4]
WAT11-*AtLUP1*	WAT11 harboring pESC-His-*AtLUP1*	This study
WAT11-*GgLUP*	WAT11 harboring pESC-His-*GgLUP*	This study
WAT11-*OeLUP*	WAT11 harboring pESC-His-*OeLUP*	This study
WAT11-*LjLUP*	WAT11 harboring pESC-His-*LjLUP*	This study
W*Oe*	WAT11 harboring pESC-Leu2d & pESC-Ura-*OeLUP*	This study
W*TOe*	WAT11 harboring pESC-Leu2d-*tSQS* & pESC-Ura-*OeLUP*	This study
W*HOe*	WAT11 harboring pESC-Leu2d-*hSQS* & pESC-Ura-*OeLUP*	This study
W*CHROe*	WAT11 harboring pESC-Leu2d-*AaCPR*-*hSQS*-*rSE* & pESC-Ura-*OeLUP*	This study
W*CHHOe*	WAT11 harboring pESC-Leu2d-*AaCPR*-*hSQS*-*hSE* & pESC-Ura-*OeLUP*	This study
EPY300	S288C, *MATα* his3△1 leu2△0 P_{GAL1}-tHMG1::δ1 P_{GAL1}-upc2-1::δ2 erg9::P_{MET3}-ERG9::HIS3 P_{GAL1}-ERG20::δ3 P_{GAL1}-tHMG1::δ4	[5]
E*Oe*	EPY300 harboring pESC-Leu2d & pESC-Ura-*OeLUP*	This study
E*TOe*	EPY300 harboring pESC-Leu2d-*tSQS* & pESC-Ura-*OeLUP*	This study
E*HOe*	EPY300 harboring pESC-Leu2d-*hSQS* & pESC-Ura-*OeLUP*	This study
E*CHROe*	EPY300 harboring pESC-Leu2d-*AaCPR*- *hSQS*-*rSE* & pESC-Ura-*OeLUP*	This study
E*CHHOe*	EPY300 harboring pESC-Leu2d-*AaCPR*-*hSQS*-*hSE* & pESC-Ura-*OeLUP*	This study

References

1. Yin H, Zhuang YB, Li EE, Bi HP, Zhou W, Liu T. Heterologous biosynthesis of costunolide in *Escherichia coli* and yield improvement. *Biotechnol. Lett.* **37**, 1249-1255 (2015).

2. Peralta-Yahya PP, Ouellet M, Chan R, Mukhopadhyay A, Keasling JD, Lee TS. Identification and microbial production of a terpene-based advanced biofuel. *Nat. Commun.* **2**, 483 (2011).

3. Ro DK, Ouellet M, Paradise EM, Burd H, Eng D, Paddon CJ, Newman JD, Keasling JD. Induction of multiple pleiotropic drug resistance genes in yeast engineered to produce an increased level of anti-malarial drug precursor, artemisinic acid. *BMC Biotechnol.* **8**, 83 (2008).

4. Urban P, Mignotte C, Kazmaier M, Delorme F, Pompon D. Cloning, yeast expression, and characterization of the coupling of two distantly related *Arabidopsis thaliana* NADPH-cytochrome P450 reductases with P450 CYP73A5. *J. Biol. Chem.* **272**, 19176-19186 (1997).

5. Nguyen TD, MacNevin G, Ro DK. *De novo* synthesis of high-value plant sesquiterpenoids in yeast. *Methods Enzymol.* **517**, 261-278 (2012).
